# The impact on renal function after long-term use of anticoagulants in atrial fibrillation patients

**DOI:** 10.1186/s12959-021-00351-1

**Published:** 2021-12-11

**Authors:** Wei-Chieh Lee, Pai-Wei Lee, Po-Jui Wu, Yen-Nan Fang, Huang-Chung Chen, Yu-Sheng Lin, Hsiu-Yu Fang, Shang-Hung Chang, Ping-Yen Liu, Mien-Cheng Chen

**Affiliations:** 1 Institute of Clinical Medicine, College of Medicine, National Cheng Kung University, Tainan, Taiwan; 2 Division of Cardiology, Department of Internal Medicine, Kaohsiung Chang Gung Memorial Hospital, Chang Gung University College of Medicine, 123 Ta Pei Road, Niao Sung District, Kaohsiung City, 83301 Taiwan; 3 Center for Big Data Analytics and Statistics, Chang-Gung University and Hospital, Taipei, Taiwan; 4 Division of Cardiology, Chang Gung Memorial Hospital, Chiayi, Taiwan

**Keywords:** Atrial fibrillation, Acute kidney injury, Estimated glomerular filtration rate, Warfarin, Non-vitamin K oral anticoagulant

## Abstract

**Objective:**

Long-term oral anticoagulant should be considered or recommended in patients with atrial fibrillation (AF) and CHA2DS2VASc score ≥ 1 for stroke prevention. Warfarin and different direct oral anticoagulants (DOACs) are metabolized differently by the kidney. The impact on renal function after long-term use of anticoagulants in the patients with AF remains unclear. This study aimed to compare DOACs and warfarin’s impact on the decline in renal function from a large cohort with AF.

**Methods:**

This study included patients with nonvalvular AF from 2000 to 2018, mainly through the medical history (ICD code) of the Chang Gung Research Database. Baseline estimated glomerular filtration rate (eGFR), follow-up eGFR and the change in eGFR between 2-year eGFR and baseline eGFR were compared between different DOACs and warfarin after propensity score matching. The primary study endpoint was acute kidney injury (AKI).

**Results:**

3657 patients were enrolled in this study and the mean observation time was 3.3 ± 0.9 years. During the observation period, there was a significantly higher incidence of AKI during follow-up in the warfarin group than in the different DOAC groups before and after propensity score matching (before: warfarin vs. DOAC: 9.2% vs. 5.2%, *p* <  0.001; after: warfarin vs. DOAC: 8.9% vs. 4.4%, *p* <  0.001). There was no difference in the incidence of AKI between dabigatran group and anti-factor Xa inhibitor group after propensity score matching. The incidence of AKI was similar among rivaroxaban, apixaban and edoxaban groups after propensity score matching. The change in eGFR between 2-year eGFR and baseline eGFR did not differ between the warfarin and DOAC groups after propensity score matching (warfarin vs. DOAC: − 1.27 ± 20.32 vs. -1.94 ± 17.24 mL/min/1.73 m^2^, *p* = 0.461).

**Conclusions:**

During the mean observation time of 3.3 ± 0.9 years, warfarin was associated with a higher incidence of AKI compared with DOACs. The decline in renal function did not differ among warfarin and different DOAC groups.

**Supplementary Information:**

The online version contains supplementary material available at 10.1186/s12959-021-00351-1.

## Background

Due to the aging population, increasing number of patients experiences atrial fibrillation (AF). Long-term oral anticoagulant should be considered in AF patients with CHA2DS2VASc score = 1 and is strongly recommended in patients with CHA2DS2VASc score ≥ 2 for stroke prevention. Warfarin has been reported to cause arterial calcification and microthrombus, which contribute to worsening renal function in warfarin users [1, 2]. Direct oral anticoagulants (DOACs) have predictable anticoagulant effects, infrequent monitoring requirements and less drug-food interactions compared to warfarin [3]. Moreover, warfarin and different DOACs are metabolized differently by the kidney [3]. The optimal regimen for patients with chronic kidney disease (CKD) needing anticoagulants is currently debated [4]. In the Randomized Evaluation of Long-Term Anticoagulation Therapy (RE-LY) study, after an average of 30 months, AF patients taking warfarin exhibited a greater decline in renal function compared with those taking dabigatran (warfarin vs. dabigatran 150 mg vs. dabigatran 110 mg; − 3.68 ± 0.24 vs. –2.46 ± 0.23 vs. –2.57 ± 0.24 ml/min; *p* = 0.0002 and *p* = 0.0009, respectively) [5]. However, there was a small, statistically significant decline in creatinine clearance (CrCl) among patients receiving rivaroxaban compared with patients receiving warfarin in the subgroup analysis of ROCKET-AF trial (Rivaroxaban Once Daily Oral Direct Factor Xa Inhibition Compared with Vitamin K Antagonism for Prevention of Stroke and Embolism Trial in Atrial Fibrillation) (warfarin vs. rivaroxaban; − 3.5 ± 15.1 vs. − 4.3 ± 14.6 mL/min; *P* <  0.001) [6, 7]. On the contrary, in the US medical care database, rivaroxaban was associated with a 19% reduction in the hazard of acute kidney injury (AKI) and an 18% reduction in progression to stage 5 CKD or hemodialysis compared with warfarin in patients with AF [8]. Moreover, in a recent multicenter prospective cohort study, patients taking direct oral anticoagulants (DOACs) showed a slower decline in renal function compared with those taking warfarin, but the favorable association between DOAC use and decline of renal function was partially lost in patients with diabetes [9]. Thus, there are different impacts of DOACs and warfarin on renal decline in different population with AF. Moreover, there are limited data in terms of different impacts on renal decline of long-term use of the four different DOACs and warfarin in AF patients in the Asian population [10].

According, we conducted this study to compare DOACs and warfarin’s impact on the decline in renal function from a large Chang Gung Research Database (CGRD), which had detailed laboratory data for comparison.

## Methods

### Patient population

This study included patients with AF from January 2000 to December 2018, mainly through the medical history obtained from the CGRD. The CGRD is based on the largest healthcare system in Taiwan, which comprises four tertiary care medical centers and three major teaching hospitals with a total of 10,050 beds [11]. The CGRD contains data of detailed laboratory values and drug use.

The inclusion criteria were as follows: patients aged ≥18 years and diagnosed with AF (International Classification of Diseases, Ninth Revision, Clinical Modification (ICD-9-CM) code 427.31 or Tenth Revision (ICD-10) codes I480, I481, I482, and I4891), and patients who were prescribed the same oral anticoagulant (warfarin or DOACs) for more than 2 years. All patients in the DOAC groups had their dosage adjustment according to dosing criteria of each DOAC, especially while renal function declined.

**Fig. 1:**
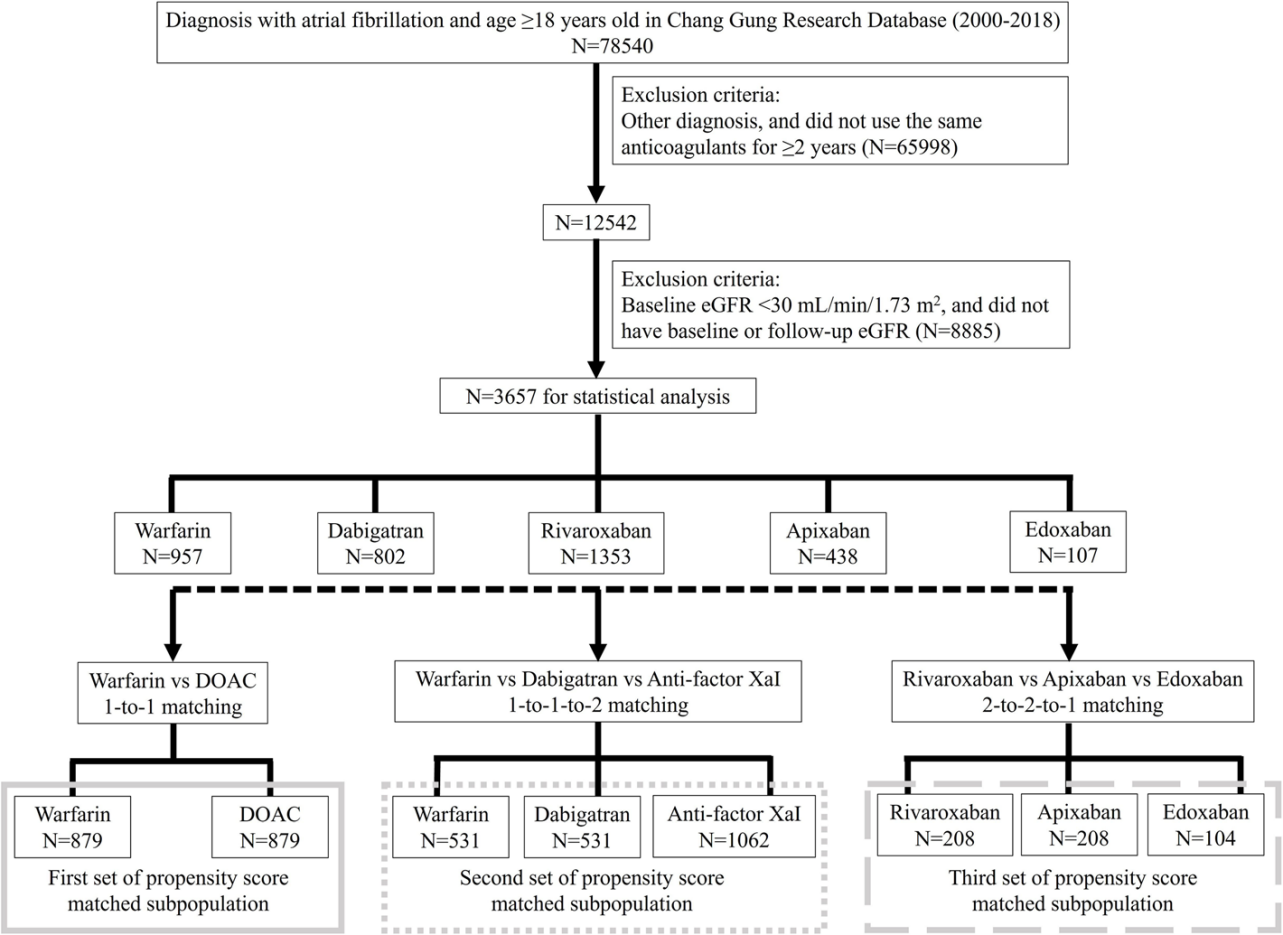
Flow chart of the study population eGFR: estimated glomerular filtration rate; DOAC: direct oral anticoagulants.

Exclusion criteria were as follows: patients who had taken oral anticoagulants for mechanical valves, rheumatic mitral stenosis, pulmonary embolism, venous thromboembolism, or preventive use after orthopedic surgery and patients with baseline estimated glomerular filtration rate (eGFR) < 30 mL/min. The flowchart of the study population is illustrated in Fig. 1.

Data on general demographics, comorbidities, baseline, and follow-up eGFR, medication use, presence of AKI, the need for hemodialysis, and renal death were obtained and compared between the warfarin and DOAC groups, among warfarin, dabigatran, and anti-factor Xa inhibitors, and between different anti-factor Xa inhibitors.

The patients and the public did not involve in the design of this study but involved in the process of review of institutional review committee.

### Ethical statement

This retrospective study conformed to the ethical guidelines of the 1975 Declaration of Helsinki and was approved for human research by the institutional review committee of Kaohsiung Chang Gung Memorial Hospital (number: 202000917B0).

### Definition

Renal function was estimated using as modification of diet in renal disease *equation*. AKI was defined as increase in serum Cr by ≥0.3 mg/dL within 48 h or increase in serum Cr to ≥1.5 times the baseline, which was known or presumed to have occurred within the past 7 days [12]. The need for hemodialysis was defined as patients presenting with oliguria or anuria and receiving hemodialysis. Renal death was defined as death due to renal disease.

### Study endpoint

The primary study endpoint was AKI, and the secondary endpoints were the need for hemodialysis and renal death.

### Statistical analysis

Data are presented as mean ± standard deviation or numbers (percentages). The clinical characteristics of the two groups were compared using the independent samples t-test or analysis of variance test for continuous variables and the chi-squared test or Fisher’s exact test for categorical variables. Propensity score matching was performed using a multivariate logistic regression model to adjust for differences in baseline characteristics (sex, age, diabetes mellitus, hypertension, hyperlipidemia, heart failure, serum Cr, eGFR, and angiotensin-converting enzyme inhibitors [ACEIs]/angiotensin-receptor blockers [ARBs]) for 1-to-1 or 1-to-1-to 2 or 2-to-2-to-1 matched analysis. Using the estimated logits, the different groups had the closest estimated logit values for comparison between different groups. Matching quality was analyzed using the absolute value of the standardized mean difference (ASMD) between the groups after matching, where a value lower than 0.1 represented negligible difference between the groups. Statistical significance was set at *p*-value < 0.05. All analyses were performed using SAS version 9.4 (SAS Institute Inc., Cary, NC, USA).

## Results

### Baseline characteristics and renal outcomes between warfarin and DOAC groups (before propensity score matching)

3657 patients were enrolled in this study and the mean observation time was 3.3 ± 0.9 years. The baseline characteristics and renal outcomes of the study population before propensity score matching are shown in Table 1. There were more male patients in the DOACs group (62.5%) compared with warfarin group (54.2%) (*p* <  0.001). Patients in the warfarin group were younger than those in the DOAC groups (warfarin vs. DOACs; 66 ± 11.3 vs. 70 ± 9.6; *p* <  0.001). Patients in the warfarin group had a lower prevalence of hypertension and hyperlipidemia, and a higher prevalence of heart failure than those in the DOAC groups. Patients in the warfarin group had a higher prevalence of CKD stage ≥3 (warfarin vs. DOACs; 37.1% vs. 24.6%; *p* = 0.009) than those in the DOAC groups. CHA2DS2-VASc scores significantly differ among warfarin and different DOAC groups (*p* <  0.001). Patients in the warfarin group had a lower prevalence of ACEI/ARB use than those in the DOAC groups. The average level of international normalized ratio (INR) in the warfarin group was 1.69 ± 0.93 at the 1-year follow-up and 1.56 ± 0.99 at the 2-year follow-up.

**Table 1:** Baseline characteristics and renal outcomes (before propensity score matching)

Variables	Warfarin group	DOAC group				*p* value (NOACs vs. Warfarin)
	Warfarin	Dabigatran	Rivaroxaban	Apixaban	Edoxaban	
N	957	802	1353	438	107	
Gender (male)	519 (54.2)	526 (65.6)	807 (59.6)	282 (64.4)	73 (68.2)	< 0.001
Mean age	66 ± 11.3	69 ± 9.6	70 ± 9.5	71 ± 9 .8	69 ± 10.0	< 0.001
Medical history						
Type 2 DM	179 (18.7)	166 (20.7)	289 (21.4)	104 (23.7)	24 (22.4)	0.065
Hypertension	346 (36.2)	336 (41.9)	625 (46.2)	230 (52.5)	50 (46.7)	< 0.001
Hyperlipidemia	184 (19.2)	171 (21.3)	303 (22.4)	126 (28.8)	35 (32.7)	0.007
Heart failure	136 (14.2)	62 (7.7)	135 (10.0)	28 (13.2)	9 (8.4)	< 0.001
Prior stroke	40 (4.2)	50 (6.2)	79 (5.8)	20 (4.6)	5 (4.7)	0.085
Vascular disease	20 (2.1)	11 (1.4)	24 (1.8)	20 (4.6)	0 (0)	1.000
Chronic kidney disease						
Stage < 3	602 (62.9)	597 (74.4)	897 (66.3)	272 (62.1)	60 (56.1)	0.009
Stage ≥3	355 (37.1)	205 (25.6)	246 (18.2)	166 (37.9)	47 (43.9)	
CHA2DS2-VASc	2.17 ± 1.33	2.42 ± 1.39	2.62 ± 1.33	2.6 2 ± 1.30	2.45 ± 1.40	< 0.001
Medication						
ACEI/ARB	528 (55.2)	472 (58.9)	803 (59.3)	254 (58.0)	72 (67.3)	0.029
Spironolactone	0 (0)	0 (0)	0 (0)	2 (0.5)	1 (0.9)	0.572
Renal function						
The average serum Cr (mg/dL)	1.09 ± 0.36	1.02 ± 0.27	1.06 ± 0.31	1.11 ± 0.34	1.14 ± 0.36	0.017
Baseline eGFR (mL/min/1.73 m^2^)	70.51 ± 24.95	74.98 ± 22.17	71.38 ± 23.38	68.51 ± 21.34	68.06 ± 21.89	0.128
1-year eGFR (mL/min/1.73 m^2^)	69.26 ± 23.86	73.33 ± 22.59	68.74 ± 22.55	66.71 ± 21.72	69.03 ± 23.49	0.553
2-year eGFR (mL/min/1.73 m^2^)	69.52 ± 26.89	72.73 ± 25.14	68.14 ± 23.01	65.80 ± 22.80	67.05 ± 22.78	0.657
Renal outcomes						
Acute kidney injury (%)	88 (9.2)	40 (5.0)	69 (5.1)	27 (6.2)	4 (3.7)	< 0.001
Renal failure requiring HD (%)	3 (0.3)	0 (0)	2 (0.1)	1 (0.2)	0 (0)	0.188
Renal death (%)	5 (0.5)	3 (0.4)	4 (0.3)	1 (0.2)	1 (0.9)	0.378
Observation time (years)	3.9 ± 1.5	3.8 ± 1.1	3.5 ± 0.8	2.9 ± 0.3	2.6 ± 0.4	< 0.001

Data were presented as mean ± standard deviation or numbers (percentages)

Abbreviation: *N* number, *DOAC* direct oral anticoagulants, *DM* diabetes mellitus, *Cr* creatinine, *eGFR* estimated glomerular filtration rate, *ACEI* Angiotensin-converting enzyme inhibitor, *ARB* angiotensin receptor blocker, *HD* hemodialysis

Although baseline serum Cr level significantly differ among warfarin and different DOAC groups (warfarin vs. DOACs; 1.09 ± 0.36 mg/dL vs. 1.06 ± 0.31 mg/dL; *p* <  0.001), baseline eGFR did not significantly differ among warfarin and different DOAC groups (warfarin vs. DOACs; 70.51 ± 24.95 mL/min vs. 71.85 ± 22.75 mL/min; *p* = 0.128). Moreover, follow-up 1-year and 2-year eGFR did not significantly differ among warfarin and different DOAC groups (Table 1). However, during the observation period, there was a significantly higher incidence of AKI in the warfarin group than in the different DOAC groups (warfarin vs. DOACs; 9.2% vs. 5.2%; *p* <  0.001). During the observation period, there were no significant differences in the need for hemodialysis and renal death among warfarin and different DOAC groups.

### Baseline characteristics and renal outcomes between warfarin and DOAC groups (after propensity score matching)

The baseline characteristics and renal outcomes of the warfarin and DOAC groups after propensity score matching are shown in Table 2. Age, gender, and the prevalence of all comorbidities were similar between warfarin and DOAC groups. The prevalence of CKD stage ≥3 and the mean CHA2DS2-VASc score did not differ between warfarin and DOAC groups. The prevalence of ACEI/ARB use was similar between warfarin and DOAC groups.

**Table 2:** Baseline characteristics and renal outcomes (after warfarin and DOACs 1:1 propensity score matching)

Variables	Warfarin group	DOAC group	*p* value	ASMD
N	879	879		
Gender (male)	490 (55.7)	502 (57.1)	0.597	0.002
Mean age	67 ± 10.6	67 ± 10.3	0.967	0.002
Medical history 1				
Type 2 DM	160 (18.2)	166 (18.9)	0.759	0.002
Hypertension	335 (38.1)	334 (38.0)	1.000	0.0002
Hyperlipidemia	172 (19.6)	181 (20.6)	0.634	0.002
Heart failure	109 (12.4)	115 (13.1)	0.721	0.002
Previous stroke	38 (4.3)	49 (5.6)	0.272	0.006
Vascular disease	19 (2.2)	16 (1.8)	0.733	0.002
Chronic kidney disease				
Stage < 3	556 (63.3)	550 (62.6)	0.805	0.001
Stage ≥3	323 (36.7)	329 (37.4)		0.001
CHA2DS2-VASc	2.23 ± 1.34	2.29 ± 1.37	0.351	0.044
Medication				
ACEI/ARB	494 (56.2)	476 (54.2)	0.415	0.003
Spironolactone	0 (0)	0 (0)	–	–
Renal function				
The average serum Cr (mg/dL)	1.08 ± 0.35	1.09 ± 0.35	0.912	0.029
Baseline eGFR (mL/min/1.73 m^2^)	70.33 ± 24.21	70.54 ± 24.67	0.861	0.009
1-year eGFR (mL/min/1.73 m^2^)	68.98 ± 23.57	69.56 ± 24.09	0.613	0.024
2-year eGFR (mL/min/1.73 m^2^)	69.06 ± 26.46	68.60 ± 24.84	0.708	0.018
Renal outcomes				
Acute kidney injury (%)	78 (8.9)	39 (4.4)	< 0.001	0.017
Renal failure requiring HD (%)	2 (0.2)	2 (0.2)	1.000	0
Renal death (%)	4 (0.5)	3 (0.3)	1.000	0.002

Data were presented as mean ± standard deviation or numbers (percentages)

Abbreviation: *DOAC* direct oral anticoagulants, *ASMD* absolute standardized mean difference, *N* number, *DM* diabetes mellitus, *Cr* creatinine, *eGFR* estimated glomerular filtration rate, *ACEI* Angiotensin-converting enzyme inhibitor, *ARB* angiotensin receptor blocker, *HD* hemodialysis

**Fig. 2:**
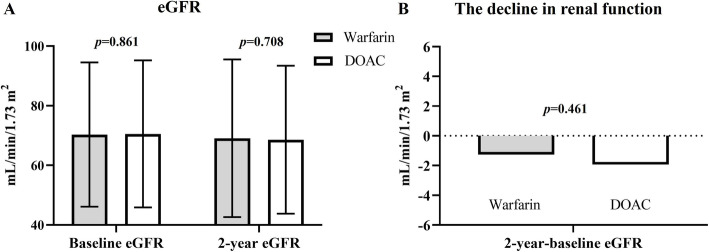
(**A**) Comparison of baseline and 2-year follow-up estimated glomerular filtration rate (eGFR) between warfarin and DOAC groups after propensity score matching. (**B**) Comparison of the change in eGFR between 2-year eGFR and baseline eGFR between warfarin and DOAC groups after propensity score matching

There was no significant difference in serum Cr level, baseline eGFR, 1-year and 2-year follow-up eGFR between the warfarin and DOAC groups after propensity score matching (Table 2) (Fig. 2A). Moreover, the change in eGFR between 2-year eGFR and baseline eGFR did not differ between warfarin and DOAC groups (warfarin vs. DOAC: − 1.27 ± 20.32 vs. -1.94 ± 17.24 mL/min/1.73 m^2^, *p* = 0.461) (Fig. 2B). In the subgroups analysis, including age ≥ 70 or < 70 years, with or without diabetes mellitus, with or without heart failure, with or without hypertension, with or without ACEI/ARB use, or CKD stage ≥3 or < 3, the change in eGFR between 2-year eGFR and baseline eGFR did not differ between warfarin and DOAC groups (Supplemental Fig. 1). However, during the observation period, there was a higher incidence of AKI during follow-up in the warfarin group than in the DOAC group (8.9% vs. 4.4%; *p* <  0.001). During the observation period, the incidence of hemodialysis and renal death did not differ between warfarin and DOAC groups.

### Baseline characteristics and renal outcomes among warfarin, dabigatran, and anti-factor Xa inhibitor groups (after propensity score matching)

The baseline characteristics and renal outcomes of warfarin, dabigatran, and anti-factor Xa inhibitor groups after propensity score matching are listed in Table 3. Patients in the dabigatran group were younger and had a lower prevalence of hypertension and CKD stage ≥3 compared with warfarin and anti-factor Xa inhibitor groups.

**Table 3:** Baseline characteristics and renal outcomes (after warfarin, dabigatran, and anti-factor Xa inhibitors 1:1:2 propensity score matching)

Variable	Warfarin	Dabigatran	Anti-factor Xa inhibitors	*p* value	ASMD		
					W vs D	W vs XaI	D vs XaI
N	531	531	1062				
Gender (male)	344 (64.8)	358 (67.4)	660 (62.1)	0.110	0.003	0.003	0.007
Mean age	71 ± 8.8^a, b^	70 ± 8.7^a^	71 ± 8.6^b^	0.004	0.108	0.068	0.177
Medical history							
Type 2 DM	111 (20.9)	104 (19.6)	238 (22.4)	0.415	0.003	0.003	0.006
Hypertension	258 (48.6)^a^	214 (40.3)^b^	524 (49.3)^a^	0.002	0.012	0.001	0.014
Hyperlipidemia	127 (23.9)	118 (22.2)	267 (25.1)	0.436	0.004	0.002	0.006
Heart failure	34 (6.4)	34 (6.4)	87 (8.2)	0.285	0	0.007	0.002
Previous stroke	30 (5.6)	31 (5.8)	58 (5.5)	0.952	0.001	0.001	0.002
Vascular disease	14 (2.6)	6 (1.1)	24 (2.3)	0.188	0.011	0.002	0.009
Chronic kidney disease							
Stage < 3	355 (66.9)^a^	407 (76.6)^b^	678 (63.8)^a^	< 0.001	0.012	0.004	0.015
Stage ≥3	176 (33.1)^a^	124 (23.4)^b^	384 (36.2)^a^		0.018	0.005	0.024
CHA2DS2-VASc	2.55 ± 1.30^a^	2.40 ± 1.33^a^	3.26 ± 1.33^b^	< 0.001	0.114	0.106	0.218
Medication							
ACEI/ARB	329 (62.0)	310 (58.4)	669 (63.0)	0.199	0.005	0.001	0.006
Spironolactone	0 (0)	0 (0)	2 (0.4)	0.500	–	0.006	0.006
Renal function							
The average serum Cr (mg/dL)	1.06 ± 0.30^a^	1.00 ± 0.26^b^	1.07 ± 0.30^a^	< 0.001	0.214	0.033	0.249
Baseline eGFR (mL/min/1.73 m^2^)	71.47 ± 22.42^a^	76.00 ± 21.91^b^	69.65 ± 21.24^a^	< 0.001	0.204	0.083	0.294
1-year eGFR (mL/min/1.73 m^2^)	68.90 ± 21.78^a^	73.85 ± 22.02^b^	67.82 ± 21.21^a^	< 0.001	0.226	0.050	0.279
2-year eGFR (mL/min/1.73 m^2^)	68.87 ± 24.74^a^	73.68 ± 25.72^b^	66.91 ± 21.82^a^	< 0.001	0.191	0.084	0.284
Renal outcomes							
Acute kidney injury (%)	54 (10.2)^a^	23 (4.3)^b^	57 (5.4)^b^	< 0.001	0.022	0.017	0.005
Renal failure requiring HD (%)	0 (0)	0 (0)	1 (0.1)	1.000	–	0.004	0.004
Renal death (%)	3 (0.6)	0 (0)	4 (0.4)	0.288	0.011	0.003	0.009

Data were presented as mean ± standard deviation or numbers (percentages)

Different letters (a, b) associated with different groups indicate significant difference (at 0.05 level) by Bonferroni multiple comparison procedure

Abbreviation: *ASMD* absolute standardized mean difference, *W* warfarin, *D* dabigatran, *XaI* anti-factor Xa inhibitor, *N* number, *DM* diabetes mellitus, *Cr* creatinine, *eGFR* estimated glomerular filtration rate, *ACEI* Angiotensin-converting enzyme inhibitor, *ARB* angiotensin receptor blocker, *HD* hemodialysis

**Fig. 3:**
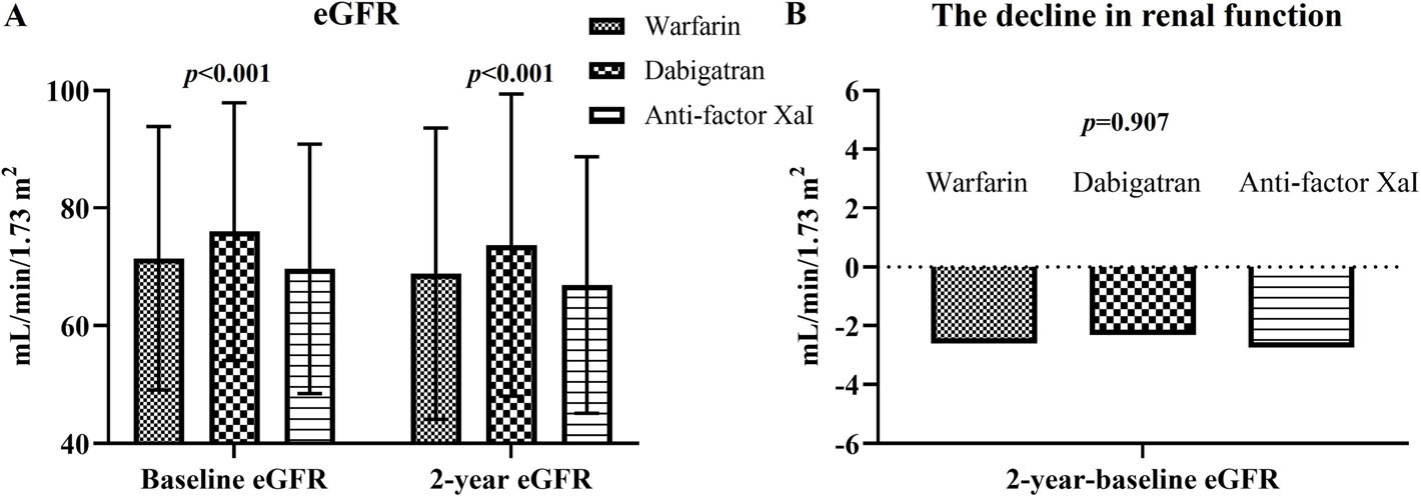
(**A**) Comparison of baseline and 2-year follow-up estimated glomerular filtration rate (eGFR) among warfarin, dabigatran, and anti-factor Xa inhibitor groups after propensity score matching. (**B**) Comparison of the change in eGFR between 2-year eGFR and baseline eGFR among warfarin, dabigatran, and anti-factor Xa inhibitor groups after propensity score matching

Patients in the dabigatran group had a lower serum Cr level and higher baseline and follow-up eGFR compared with warfarin and anti-factor Xa inhibitor groups (Fig. 3A). There was no difference in serum Cr level, and baseline and follow-up eGFR between warfarin and anti-factor Xa inhibitor groups. Of note, the change in eGFR between 2-year eGFR and baseline eGFR did not differ among dabigatran, warfarin, and anti-factor Xa inhibitor groups (Fig. 3B). In the subgroups analysis, including age ≥ 70 or < 70 years, with or without diabetes mellitus, with or without heart failure, with or without hypertension, with or without ACEI/ARB use, or CKD stage ≥3 or < 3, the change in eGFR between 2-year eGFR and baseline eGFR did not differ among dabigatran, warfarin, and anti-factor Xa inhibitor groups (Supplemental Fig. 2). However, during the observation period, the incidence of AKI was significantly higher in the warfarin group compared with dabigatran and anti-factor Xa inhibitor groups (10.2% vs. 4.3% vs. 5.4%; *p* <  0.001) (Table 3). There was no difference in the incidence of AKI between dabigatran and anti-factor Xa inhibitor groups. The incidences of hemodialysis and renal death were similar among warfarin, dabigatran, and anti-factor Xa inhibitor groups (Table 3).

### Baseline characteristics and renal outcomes among three different anti-factor Xa inhibitor groups (after propensity score matching)

The baseline characteristics and renal outcomes of rivaroxaban, apixaban and edoxaban groups after propensity score matching are listed in Table 4. Vascular disease was the only comorbidity to be significantly different among the three groups.

**Table 4:** Baseline characteristics and renal outcomes (after rivaroxaban, apixaban, and edoxaban 2:2:1 propensity score matching)

Variable	Rivaroxaban	Apixaban	Edoxaban	*p* value	ASMD		
					R vs A	R vs E	A vs E
N	208	208	104				
Gender (male)	128 (61.5)	137 (65.9)	71 (68.3)	0.447	0.005	0.008	0.003
Mean age	70 ± 10.3	70 ± 10.0	69 ± 10.0	0.967	0.004	0.026	0.030
Medical history							
Type 2 DM	49 (23.6)	49 (23.6)	23 (22.1)	0.953	0	0.003	0.003
Hypertension	101 (48.6)	101 (48.6)	47 (45.2)	0.828	0	0.005	0.005
Hyperlipidemia	57 (27.4)	57 (27.4)	34 (32.7)	0.565	0	0.010	0.010
Heart failure	19 (9.1)	13 (6.3)	9 (8.7)	0.523	0.010	0.002	0.009
Previous stroke	11 (5.3)	9 (4.3)	5 (4.8)	0.900	0.004	0.002	0.002
Vascular disease	6 (2.9)	2 (1.0)	0 (0)	0.018	0.014	0.024	0.014
Chronic kidney disease							
Stage < 3	130 (62.5)	131 (63.0)	59 (56.7)	0.527	0.001	0.007	0.008
Stage ≥3	78 (37.5)	77 (37.0)	45 (43.3)		0.001	0.009	0.010
CHA2DS2-VASc	2.59 ± 1.34	2.52 ± 1.35	2.44 ± 1.40	0.652	0.052	0.109	0.058
Medication							
ACEI/ARB	131 (63.0)	112 (53.8)	69 (66.3)	0.055	0.012	0.004	0.016
Spironolactone	0 (0)	1 (0.5)	1 (1.0)	0.160	0.010	0.014	0.006
Renal function							
The average serum Cr (mg/dL)	1.08 ± 0.32	1.10 ± 0.35	1.13 ± 0.35	0.504	0.060	0.149	0.086
Baseline eGFR (mL/min/1.73 m^2^)	70.04 ± 22.25	69.57 ± 22.49	68.55 ± 21.88	0.856	0.021	0.068	0.046
1-year eGFR (mL/min/1.73 m^2^)	67.53 ± 22.23	67.02 ± 20.75	69.60 ± 23.43	0.608	0.024	0.091	0.117
2-year eGFR (mL/min/1.73 m^2^)	67.13 ± 22.39	66.87 ± 23.40	67.37 ± 22.69	0.983	0.011	0.011	0.022
Renal outcomes							
Acute kidney injury (%)	5 (2.4)	10 (4.8)	4 (3.8)	0.423	0.013	0.008	0.005
Renal failure requiring HD (%)	1 (0.5)	0 (0)	0 (0)	0.400	0.010	0.010	–
Renal death (%)	1 (0.5)	0 (0)	1 (1.0)	0.160	0.010	0.006	0.014

Data were presented as mean ± standard deviation or numbers (percentages)

Abbreviation: *ASMD* absolute standardized mean difference, *R* rivaroxaban, *A* apixaban, *E* edoxaban, *N* number, *DM* diabetes mellitus, *Cr* creatinine, *eGFR* estimated glomerular filtration rate, *ACEI* Angiotensin-converting enzyme inhibitor, *ARB* angiotensin receptor blocker, *HD* hemodialysis

**Fig. 4:**
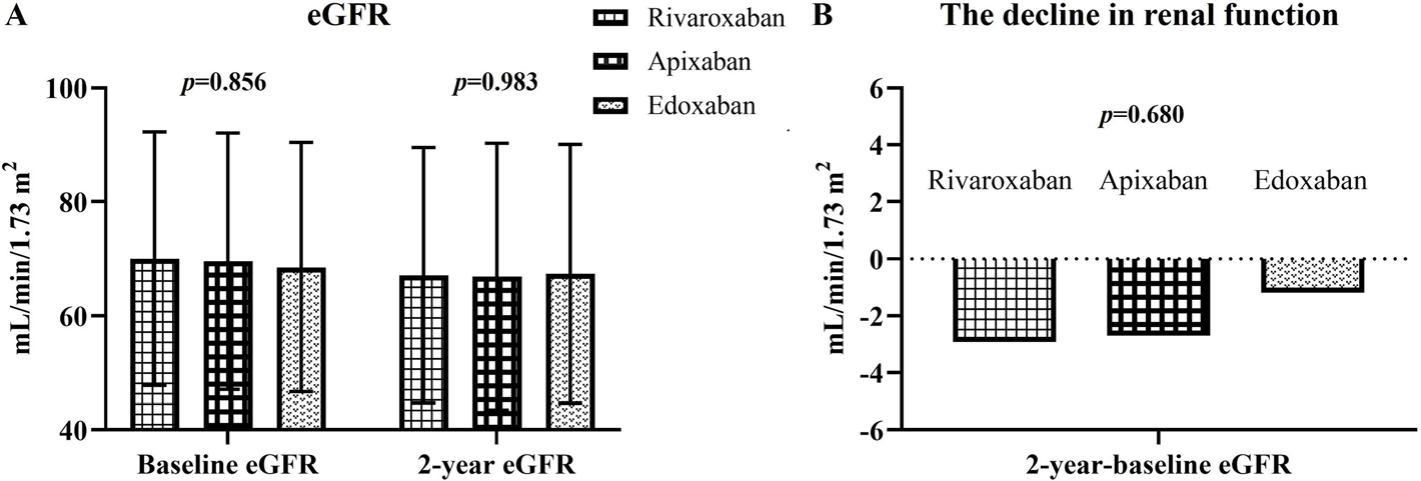
(**A**) Comparison of baseline and 2-year follow-up estimated glomerular filtration rate (eGFR) among rivaroxaban, apixaban, and edoxaban groups after propensity score matching. (**B**) Comparison of the change in eGFR between 2-year eGFR and baseline eGFR among rivaroxaban, apixaban, and edoxaban groups after propensity score matching

There was no significant difference in baseline eGFR, 1-year and 2-year follow-up eGFR among rivaroxaban, apixaban and edoxaban groups after propensity score matching (Table 4) (Fig. 4A). Moreover, the change in eGFR between 2-year eGFR and baseline eGFR did not differ among rivaroxaban, apixaban and edoxaban groups (Fig. 4B). In the subgroups analysis, including age ≥ 70 or < 70 years, with or without diabetes mellitus, with or without heart failure, with or without hypertension, with or without ACEI/ARB use, or CKD stage ≥3 or < 3, the change in eGFR between 2-year eGFR and baseline eGFR did not differ among rivaroxaban, apixaban and edoxaban groups (Supplemental Fig. 3). During the observation period, the incidence of AKI, hemodialysis, and renal death were similar among rivaroxaban, apixaban and edoxaban groups (Table 4).

## Discussion

### The significance of this study

As the frequency of anticoagulation use is increasing due to increasing number of patients experiencing AF and the dosages of DOACs during the follow-up period might need to be adjusted according to renal function, the issue of renal function decline or acute kidney injury associated with anticoagulant use in AF patients is very important and clinically relevant. In this study, during the observation period, warfarin group had a higher incidence of AKI compared with different DOAC groups before and after propensity score matching. However, the incidence of the need for hemodialysis and renal death did not differ among warfarin group and different DOACs groups. The incidences of AKI, hemodialysis and renal death were similar between dabigatran, and anti-factor Xa inhibitor groups. The incidence of AKI, hemodialysis, and renal death were similar among rivaroxaban, apixaban and edoxaban groups. The change in eGFR between 2-year eGFR and baseline eGFR did not differ between the warfarin and DOAC groups during the observation period.

### Anticoagulant-related nephropathy

For a long time, warfarin has been widely used as an anticoagulant therapy to prevent primary and secondary thromboembolic events in patients with AF. However, labile INR has been frequently observed in clinical practice. Adverse effects of warfarin therapy on the kidney have been reported and warfarin-related nephropathy is defined as unexplained AKI and hematuria (visible or nonvisible) while receiving warfarin therapy [13, 14]. Warfarin-related nephropathy is a significant risk factor for the progression of CKD and mortality [15]. Many case reports have mentioned that DOACs (dabigatran, apixaban, or rivaroxaban) could also cause AKI due to the induction of tubular red blood cell (RBC) casts and tubular necrosis or interstitial nephritis [16–18]. Therefore, all anticoagulants can cause anticoagulant-related nephropathy (ARN). Pathological findings of ARN revealed the presence of RBCs in Bowman’s space and in tubules and occlusive RBC casts predominantly in distal nephron segments with dysmorphic RBCs in the glomerulus on electron microscopy, implying injury to the glomerular filtration barrier [19]. Accordingly, the main pathological mechanism of ARN was attributed to hemorrhage in the glomerulus and tubular obstruction by RBC. Chan et al. reported that apixaban, dabigatran, and rivaroxaban were associated with a lower risk of AKI than warfarin in a large cohort of Taiwanese patients who did or did not have CKD [10]. However, they did not provide detailed serial follow-up eGFR values under different anticoagulants treatment due to the lack of laboratory data in that registry database, and the diagnoses of AKI and CKD were made according to the ICD code. Moreover, they did not provide information in terms of impact of edoxaban on the decline in renal function in AF patients. In our study, edoxaban group had a significantly lower incidence of AKI compared with warfarin group and had a similar incidence of AKI compared with rivaroxaban and, apixaban groups.

The AKI period represents the time window wherein critical interventions might be initiated to alter the natural history of kidney disease [20]. Therefore, it is possible to observe recovery of renal function with critical intervention after AKI. Moreover, the association between AKI and subsequent renal function decline is amplified by pre-existing severity of CKD [21]. In our study, patients with advanced stage of CKD (eGFR < 30 ml/min) was excluded and more than 60% of patients with CKD were in stage < 3 before and after propensity score matching. In this study, we observed that during the observation period, the change in eGFR between 2-year eGFR and baseline eGFR did not differ among warfarin, dabigatran, and anti-factor Xa inhibitor groups.

### Anticoagulants use and the decline in renal function

The decline of renal function is common in patients with AF, regardless of treatment with warfarin or DOACs. In the analysis from the RE-LY study, dabigatran may provide slower progression of renal function when compared with warfarin use [5]. There was a small, statistically significant decline in renal function among patients receiving rivaroxaban compared with patients receiving warfarin in the subgroup analysis of ROCKET-AF trial [6, 7]. In a recent multicenter prospective cohort study, Pastori et al. reported that DOACs provided a slower decline in renal function compared to those using warfarin [9]. Therefore, the results about the decline in renal function after long-term anticoagulants use is still controversial and the underlying mechanism is not clear.

In our study, we still noted a higher incidence of AKI in patients with AF using warfarin when compared those using DOACs. However, the decline in renal function did not differ between warfarin and DOAC use during the observation period. Randomized studies are warranted to clarify the issues of the association of long-term anticoagulants use and renal function decline in AF patients treated with different anticoagulants. Despite the observed trends in AKI incidence and eGFR decline, anticoagulation for stroke prevention in AF patients remains indicated because the benefits outweigh the risks.

### Study limitations

The first limitation of this study was its retrospective nature. Second, this is not a randomized study, and this study still has selective bias even though propensity score matching was performed. Third, the ICD-9 M and ICD-10 M codes were relied on each physician’s choice in clinical practice. However, we used laboratory data in the database to confirm the diagnosis of AKI and the decline in renal function. Fourth, we could not totally exclude or explore all renal toxic materials during the follow-up period. Some confounders including more hospitalization, progression of diabetes, hypertension management beyond ACEI/ARB, or progression of HF could influence the results, but were not available in our study. Finally, we only enrolled patients using the same anticoagulants for 2 years, who also had regular follow-ups of renal function during the observation period. Therefore, only a limited number of patients were finally enrolled in this study. However, this study provided the incidence of AKI and the changes in eGFR under the same anticoagulant during the long observation period. Further large prospective studies are warranted to validate our findings.

## Conclusions

During the mean observation time of 3.3 ± 0.9 years, warfarin was associated with a higher incidence of AKI compared with DOACs. The decline in renal function did not differ among warfarin and different DOAC groups.

## Supplementary Information

**Additional file 1.** Supplemental Fig. 1 Comparison of the change in estimated glomerular filtration rate (eGFR) between 2-year eGFR and baseline eGFR between warfarin and DOAC groups after propensity score matching. In the subgroups analyses, including age ≥ 70 (A) or < 70 years old (B), with (C) or without (D) diabetes mellitus (DM), with (E) or without (F) heart failure (HF), with (G) or without (H) hypertension (HTN), with (I) or without (J) angiotensin-converting enzyme inhibitor (ACEI)/angiotensin-receptor blocker (ARB) and chronic kidney disease (CKD) stage ≥3 (K) or < 3 (L). ACE: angiotensin-converting enzyme inhibitor. ARB: angiotensin-receptor blocker. Supplemental Fig. 2 Comparison of the change in estimated glomerular filtration rate (eGFR) between 2-year eGFR and baseline eGFR among warfarin, dabigatran, and anti-factor Xa inhibitor groups after propensity score matching. In the subgroups analyses, including age ≥ 70 (A) or < 70 years old (B), with (C) or without (D) diabetes mellitus (DM), with (E) or without (F) heart failure (HF), with (G) or without (H) hypertension (HTN), with (I) or without (J) angiotensin-converting enzyme inhibitor (ACEI)/angiotensin-receptor blocker (ARB) and chronic kidney disease (CKD) stage ≥3 (K) or < 3 (L). Supplemental Fig. 3 Comparison of the change in estimated glomerular filtration rate (eGFR) between 2-year eGFR and baseline eGFR among rivaroxaban, apixaban and edoxaban groups. In the subgroups analyses, including age ≥ 70 (A) or < 70 years old (B), with (C) or without (D) diabetes mellitus (DM), with (E) or without (F) heart failure (HF), with (G) or without (H) hypertension (HTN), with (I) or without (J) angiotensin-converting enzyme inhibitor (ACEI)/angiotensin-receptor blocker (ARB) and chronic kidney disease (CKD) stage ≥3 (K) or < 3 (L).

## Data Availability

The study data are *available* from the *corresponding author upon reasonable request.* This study was based in part on data from the CGRD provided by Chang Gung Memorial Hospital. The interpretation and conclusions contained herein do not represent the position of Chang Gung Memorial Hospital.

## Abbreviations

AF: atrial fibrillation
DOAC: direct oral anticoagulant
CrCl: creatinine clearance
RE-LY: Randomized Evaluation of Long-Term Anticoagulation Therapy
ROCKET-AF: Rivaroxaban Once Daily Oral Direct Factor Xa Inhibition Compared with Vitamin K Antagonism for Prevention of Stroke and Embolism Trial in Atrial Fibrillation
AKI: acute kidney injury
CGRD: Chang Gung Research Database
ICD: International Classification of Diseases
CM: Clinical Modification
eGFR: estimated glomerular filtration rate
ACEI: angiotensin-converting enzyme inhibitor
ARB: angiotensin-receptor blocker
ASMD: absolute value of the standardized mean difference
CKD: chronic kidney disease
INR: international normalized ratio
RBC: red blood cell
ARN: anticoagulant-related nephropathy
