# Correction to: The impact on renal function after longterm use of anticoagulants in atrial fibrillation patients

**DOI:** 10.1186/s12959-021-00361-z

**Published:** 2021-12-30

**Authors:** Wei-Chieh Lee, Pai-Wei Lee, Po-Jui Wu, Yen-Nan Fang, Huang-Chung Chen, Yu-Sheng Lin, Hsiu-Yu Fang, Shang-Hung Chang, Ping-Yen Liu, Mien-Cheng Chen

**Affiliations:** 1 Institute of Clinical Medicine, College of Medicine, National Cheng Kung University, Tainan, Taiwan; 2 Division of Cardiology, Department of Internal Medicine, Kaohsiung Chang Gung Memorial Hospital, Chang Gung University College of Medicine, 123 Ta Pei Road, Niao Sung District, Kaohsiung City, 83301 Taiwan; 3 Center for Big Data Analytics and Statistics, Chang-Gung University and Hospital, Taipei, Taiwan; 4 Division of Cardiology, Chang Gung Memorial Hospital, Chiayi, Taiwan

**Correction to: Thrombosis J 19, 98 (2021)**

**https://doi.org/10.1186/s12959-021-00351-1**

Following publication of the original article [1], an error was reported in the authorship list. Wei-Chieh Lee was reported as the corresponding author, however Ping-Yen Liu and Mien-Cheng Chen are the correct co-corresponding authors as shown in this Correction article.

The original article [1] has been corrected.
