# Supplementary figures and images for: The impact on renal function after long-term use of anticoagulants in atrial fibrillation patients
Source: Thromb J. 2021 Dec 11;19:98. doi: 10.1186/s12959-021-00351-1 (PMC8666070; doi:10.1186/s12959-021-00351-1)

## Supplemental figures

Supplemental Figure 1.

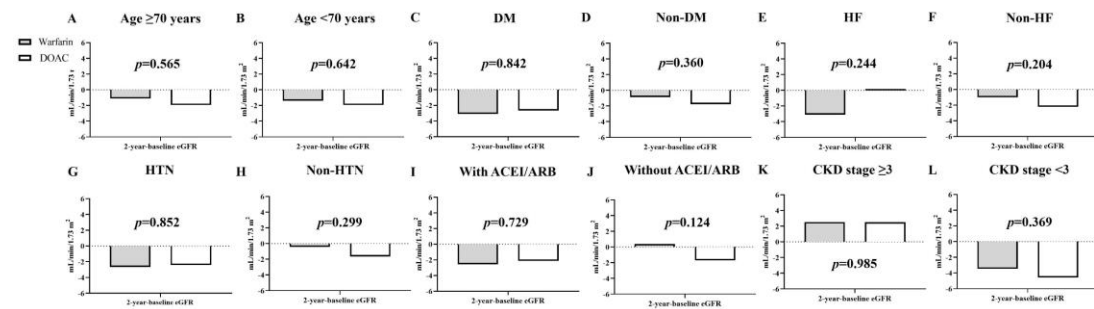

Supplemental Figure 2.

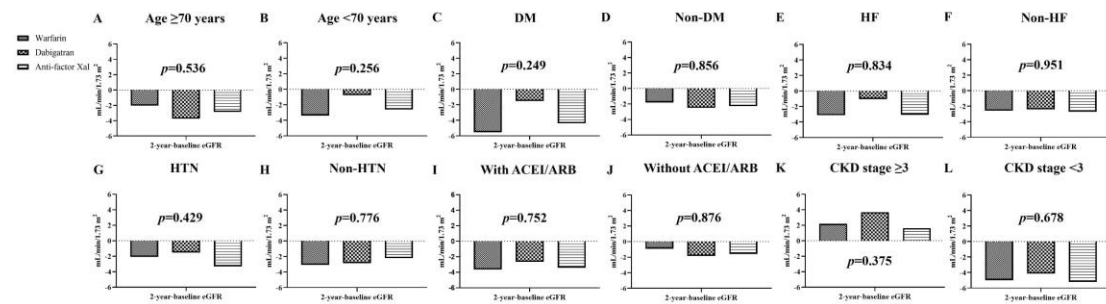

Supplemental Figure 3.

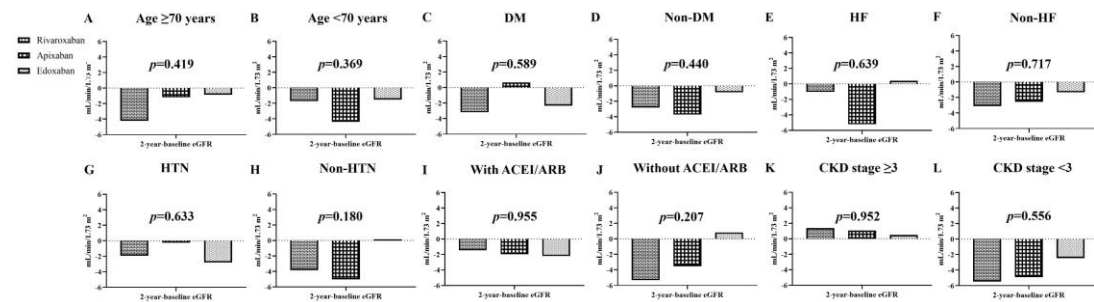

Supplement: Supplementary file 1 — Additional file 1. Supplemental Fig. 1 Comparison of the change in estimated glomerular filtration rate (eGFR) between 2-year eGFR and baseline eGFR between warfarin and DOAC groups after propensity score matching. In the subgroups analyses, including age ≥ 70 (A) or < 70 years old (B), with (C) or without (D) diabetes mellitus (DM), with (E) or without (F) heart failure (HF), with (G) or without (H) hypertension (HTN), with (I) or without (J) angiotensin-converting enzyme inhibitor (ACEI)/angiotensin-receptor blocker (ARB) and chronic kidney disease (CKD) stage ≥3 (K) or < 3 (L). ACE: angiotensin-converting enzyme inhibitor. ARB: angiotensin-receptor blocker. Supplemental Fig. 2 Comparison of the change in estimated glomerular filtration rate (eGFR) between 2-year eGFR and baseline eGFR among warfarin, dabigatran, and anti-factor Xa inhibitor groups after propensity score matching. In the subgroups analyses, including age ≥ 70 (A) or < 70 years old (B), with (C) or without (D) diabetes mellitus (DM), with (E) or without (F) heart failure (HF), with (G) or without (H) hypertension (HTN), with (I) or without (J) angiotensin-converting enzyme inhibitor (ACEI)/angiotensin-receptor blocker (ARB) and chronic kidney disease (CKD) stage ≥3 (K) or < 3 (L). Supplemental Fig. 3 Comparison of the change in estimated glomerular filtration rate (eGFR) between 2-year eGFR and baseline eGFR among rivaroxaban, apixaban and edoxaban groups. In the subgroups analyses, including age ≥ 70 (A) or < 70 years old (B), with (C) or without (D) diabetes mellitus (DM), with (E) or without (F) heart failure (HF), with (G) or without (H) hypertension (HTN), with (I) or without (J) angiotensin-converting enzyme inhibitor (ACEI)/angiotensin-receptor blocker (ARB) and chronic kidney disease (CKD) stage ≥3 (K) or < 3 (L). [file 12959_2021_351_MOESM1_ESM.pdf]
